# Digital Metastases of Giant Cell Rich Malignant Fibrous Histiocytoma

**DOI:** 10.1080/13577149977596

**Published:** 1999-12

**Authors:** Harry C. Brownlow, George Ioannidis, Max C. L. M. H. Gibbons, Adrian C. Jones, Nick Athanasou

**Affiliations:** 1 Nuffield Department of Orthopaedic Surgery Nuffield Orthopaedic Centre Headington Oxford OX3 7LD UK; 2 Churchill Hospital Oxford UK

## Abstract

*Background.* Metastatic spread of soft tissue sarcomas to the digits is extremely rare and metastasis of MFH to the fingers and toes has not been documented.

*Purpose.* We present two case reports of metastatic spread from a giant cell rich malignant fibrous histiocytoma to the digits and discuss their management.


*Sarcoma* (1999) **3**, 167–170

ORIGINAL ARTICLE

### Digital metastases of giant cell rich malignant fibrous histiocytoma

HARRY C. BROWNLOW,[1] GEORGE IOANNIDIS,[1] MAX C.L.M.H.GIBBONS,[1] ADRIAN C. JONES[2] & NICK ATHANASOU[1]

[1]*Nuffield Orthopaedic Centre,* [2]*Churchill Hospital, Oxford, UK*

**Abstract**
*Background.* Metastatic spread of soft tissue sarcomas to the digits is extremely rare and metastasis of MFH to the fingers and toes has not been documented.
*Purpose.* We present two case reports of metastatic spread from a giant cell rich malignant fibrous histiocytoma to the digits and discuss their management.

### Introduction

Malignant fibrous histiocytoma (MFH) of soft tissue is the most common soft tissue sarcoma of late adult life.[1] Metastases of MFH preferentially spread to the lungs but also to lymph nodes and the soft tissues.[1] Metastatic spread of soft tissue sarcomas to the digits is extremely rare[2] and metastasis of MFH to the fingers and toes has not been documented. In this report we present two cases of metastatic spread from a giant cell rich MFH (giant cell tumour of soft parts) to the digits.

### Case report 1

A 56-year-old man presented with a 6-week history of a rapidly growing painful lump under the nail of the right fourth toe. Thirteen months earlier he had undergone excision of a malignant fibrous histiocytoma (giant cell rich) from the anterior compartment of the contralateral lower leg. At that time staging studies demonstrated no evidence of bone involvement or pulmonary metastasis. Six months after excision of the tumour he developed haemoptysis with multiple pulmonary metastases for which he had palliative chemotherapy (three cycles of adriamycin and cisplatin).

Examination showed the tip of the toe to be expanded and ulcerated and that the toenail had been destroyed. Radiographic examination revealed a soft tissue mass with almost complete erosion of the distal phalanx (Fig. 1). The features were consistent with those of a malignant tumour.

He underwent amputation of the toe through the metatarsophalangeal joint. Pathological examination

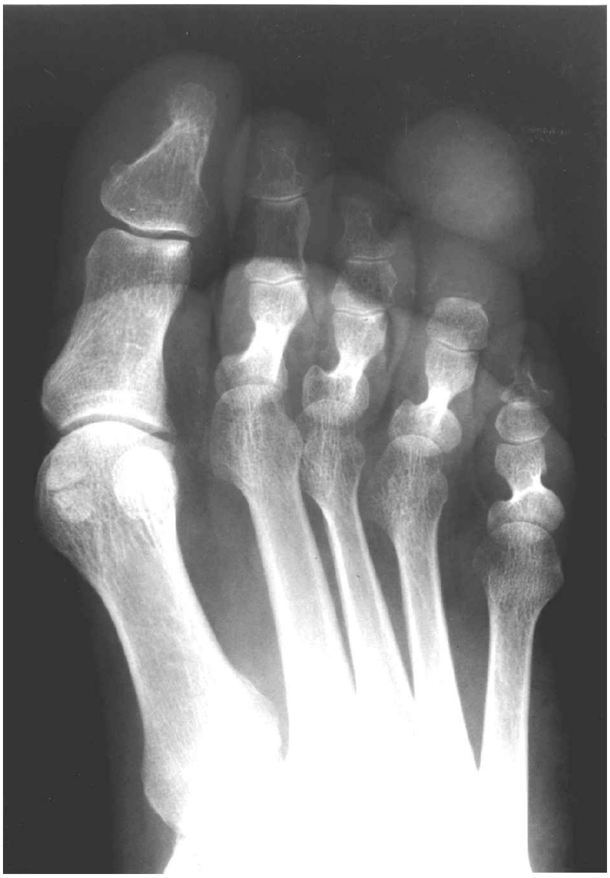

**Figure 1.** *Radiograph demonstrating destruction of the distal phalanx of the fourth toe with a large associated soft tissue mass.*

of the digit revealed a highly vascular tumour containing areas of necrosis and haemorrhage. The tumour was composed of mononuclear tumour cells

Correspondence to: Mr Harry C. Brownlow, Clinical Lecturer, Nuffield Department of Orthopaedic Surgery, Nuffield Orthopaedic Centre, Headington, Oxford OX3 7LD, UK. Tel: 01865 741155; Fax: 01865 227354; E-mail: harry.brownlow@ndos.ox.ac.uk

1357-714X print/1369-1643 online/99/040167-04 © 1999 Taylor & Francis Ltd and multinucleated osteoclast-like giant cells (Fig. 2a) The mononuclear tumour cells were large, round, polygonal or spindle-shaped, and had large vesicular or hyperchromatic nuclei. These tumour cells showed cellular and nuclear pleomorphism and contained numerous mitotic figures, some of which were atypical. The osteoclast-like giant cells contained a variable number of nuclei but did not show mitotic activity; individual nuclei frequently contained a prominent nucleolus. The giant cells and scattered mononuclear cells were positive for CD68. The tumour cells were positive for vimentin but negative for epithelial markers (cytokeratin, and epithelial membrane antigen) and desmin. They were also negative for S100 and melanoma associated antigen. Solid sheets and lobules of giant cell rich tumour tissue had completely replaced the distal phalanx and spread into the surrounding subcutaneous tissue and skin (Fig. 2b). There was no evidence of tumour bone formation. Focally, the tumour showed a storiform pattern. The tumour was diagnosed as a metastatic giant cell rich malignant fibrous histiocytoma whose features were similar to those of the primary tumour.

Over the next 5 months he presented on three occasions with three new metastases involving the left great toe (Fig. 3), the right fifth toe and the sole of the right foot. Each of these lesions required excision and pathological examination of each

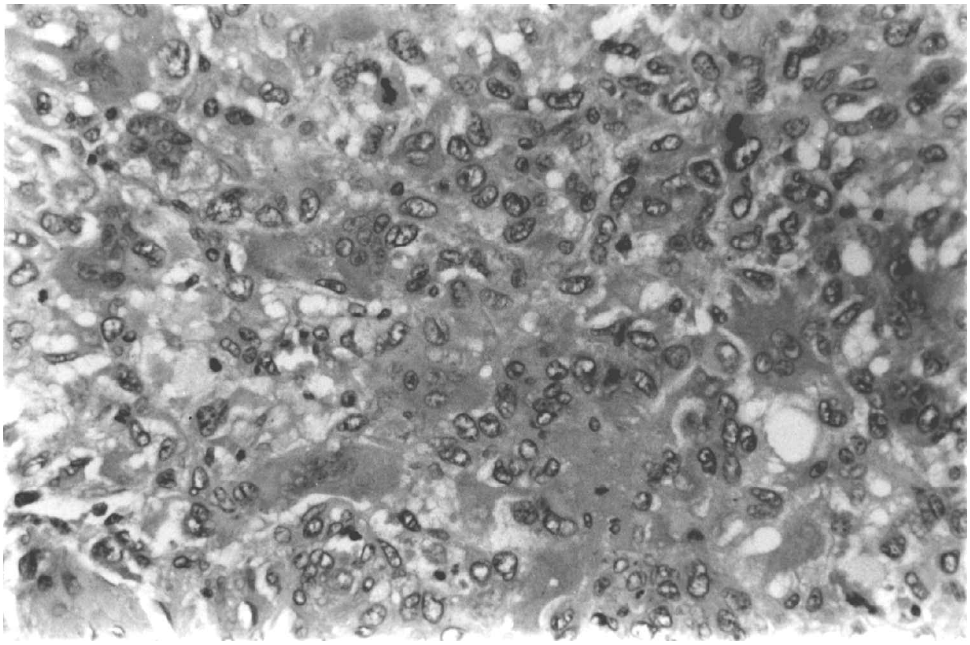

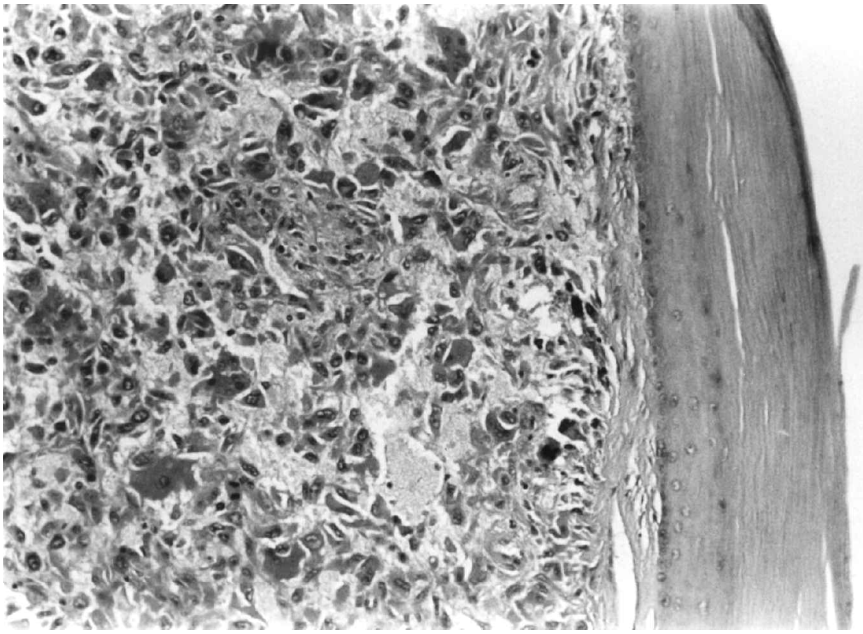

**Figure 2.** *Photomicrographs of the giant cell-rich tumour in case 1. (a) The tumour contains numerous osteoclast-like giant cells which contain a variable number of nuclei. The mononuclear cells show mitotic activity. (Haemotoxylin-eosin, ×400: original magnification.) (b) The tumour has extended up to the epidermis and contains numerous highly pleomorphic mononuclear cells some of which show mitotic activity. There are also scattered giant cells. (Haemotoxylin-eosin, ×100: original magnification.)*

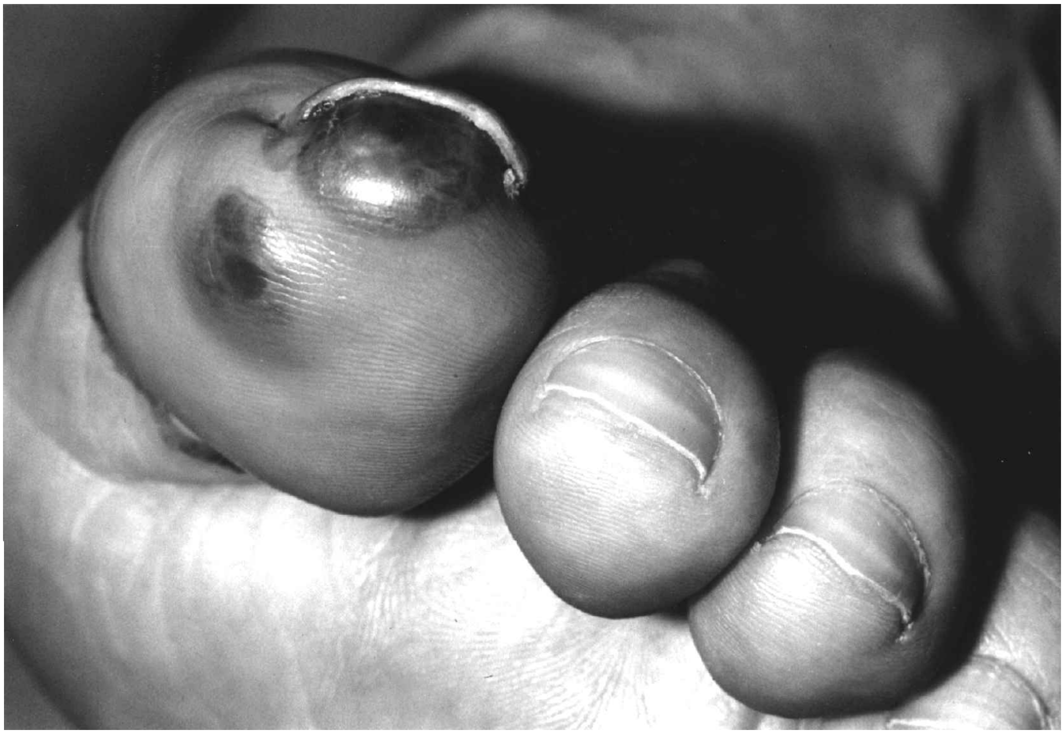

**Figure 3.** *The clinical appearance of the MFH metastasis in the great toe.*

demonstrated giant cell rich malignant fibrous histiocytoma. All the wounds healed and he retained good function—being able to walk 1 mile a day without loss of stability. He died 1 month after the last excision.

### Case report 2

A 61-year-old man presented with a 1-month history of a rapidly growing lesion in the right (dominant) long finger. Twelve months previously he had undergone excision of a 30-cm diameter MFH (giant cell rich) which was arising from the soft tissues of the posterior wall of the chest but without involvement of the ribs or scapula. At that time he already had pulmonary metastases with haemoptysis and was commenced on palliative chemotherapy (adriamycin, six cycles). Two episodes of external beam radiotherapy to the digit failed to slow the growth of the soft tissue mass which was thought to be a metastasis.

Examination revealed a 1-cm diameter nodule within the pulp of the finger with associated swelling extending to the level of the proximal interphalangeal joint.

He underwent amputation of the finger through the metacarpophalangeal joint in order to remove the lesion with clear margins. The wound healed uneventfully and he retained good function of the hand, being able to drive and operate a computer. He completed a nine-cycle course of ifosfamide and required excision of a soft tissue metastasis from the plane between the right gluteus medius and maximus. He died 10 months after the finger amputation.

Pathological examination of the digit revealed a discrete deposit in the pulp that contained areas of necrosis and haemorrhage. It was composed of highly pleomorphic tumour cells with large abnormal hyperchromatic nuclei, together with tumour giant cells and numerous multinucleated osteoclast-like giant cells which were CD68 positive. Tumour cells were positive for vimentin but negative for desmin, S100, epithelial membrane antigen, cytokeratin and melanoma associated antigen. The tumour was diagnosed as a metastatic giant cell rich malignant fibrous histiocytoma. Its features were similar to those of the primary tumour.

### Discussion

Malignant fibrous histiocytoma of soft tissues is described as having several subtypes. The giant cell rich variant is less common than the storiform-pleomorphic and myxoid types and its peak presentation is in early adulthood.[3] When this tumour arises from tissues deep to the superficial fascia it develops widespread metastases in 75% of cases. Eighty per cent of patients with pulmonary or widespread metastases die within 2 years of the date of diagnosis.[4] The treatment advocated for this aggressive soft tissue tumour is radical surgical excision[1,3,4] with chemotherapy or radiotherapy for palliation.[1,4]

Primary MFH of the digits is extremely rare[5–7] and metastases of MFH to the digits has not been described. We have performed four digital amputations (two patients) in order to gain local control of symptomatic metastases in fingers and toes. All the amputations were performed through the metacarpo- or metatarso-phalangeal joints in order to ensure resection of the tumour with clear margins and to allow wound closure with healthy tissue. The amputations were well tolerated, the wounds healed well, and the patients suffered little functional impairment.

### References

1 Rooser B, Willen H, Gustafson P, Alvegard TA, Rydholm A. Malignant fibrous histiocytoma of soft tissue. *Cancer* 1991;67:499–505.
2 Wu KK. Bronchogenic carcinoma with metastases to the foot: a report of two cases. *J Foot Ankle Surg* 1995;34:322–6.
3 Enzinger FM, Weiss SW. *Soft Tissue Tumours*. St. Louis: Mosby, 1995.
4 Guccion JG, Enzinger FM. Malignant giant cell tumor of soft parts. *Cancer* 1972;29:1518–29.
5 Patel MR, Nashed R, Vigorito V, Kim B. Soft tissue malignant fibrous histiocytoma in a finger. *J Hand Surg* 1993;18A:135–7.
6 Hankin FM, Hankin RC, Louis D. Malignant fibrous histiocytoma involving a digit. *J Hand Surg* 1987;12A:83–6.
7 Bullon A, Nistal M, Razquin S, Novo A, Fregenal J, Regadera J. Malignant fibrous histiocytoma in a child's hand. *J Hand Surg* 1986;11A:744–7.
